# Functional relationship between high mobility group A1 (HMGA1) protein and insulin-like growth factor-binding protein 3 (IGFBP-3) in human chondrocytes

**DOI:** 10.1186/ar4045

**Published:** 2012-10-04

**Authors:** Giorgio Gasparini, Marco De Gori, Francesco Paonessa, Eusebio Chiefari, Antonio Brunetti, Olimpio Galasso

**Affiliations:** 1 Department of Orthopedics, Magna Græcia University, V.le Europa, Catanzaro, 88100 Italy; 2 Department of Neuroscience and Brain Technologies, Istituto Italiano di Tecnologia, Via Morego, Genoa, 16163 Italy; 3 Department of Health Sciences, Magna Græcia University, V.le Europa, Catanzaro, 88100 Italy

## Abstract

**Introduction:**

Insulin-like growth factor I (IGF-I) regulates articular cartilage homeostasis. During osteoarthritis (OA), the anabolic responses of chondrocytes to IGF-I are likely to be prevented by the enhanced production of IGF-binding proteins (IGFBPs), especially IGFBP-3. The aim of this study is to evaluate whether the architectural transcription factor high mobility group A1 (HMGA1) influences IGFBP-3 overexpression in vitro, in cultured chondrocytic cell lines, and ex vivo, in human osteoarthritic cartilage compared to healthy human cartilage controls.

**Methods:**

Quantitative real-time reverse transcription-PCR (qRT-PCR) was performed to assess the relative transcript levels of HMGA1 and IGFBP-3 in vitro, in the human chondrocytic cell lines T/C-28a4 and C-28/I2. An electrophoretic mobility shift assay (EMSA), chromatin immunoprecipitation (ChIP) and transient transfection assays were performed to investigate the HMGA1-*IGFBP-3 *gene interaction. Samples of articular cartilage were harvested from osteoarthritic patients and controls and analyzed by qRT-PCR for HMGA1 and IGFBP-3 mRNA levels.

**Results:**

A parallelism between HMGA1 protein levels and *IGFBP-3 *gene expression has been observed in T/C-28a4 and C-28/I2 cells. The interaction of HMGA1 with the *IGFBP-3 *gene promoter has been demonstrated by EMSA and ChIP. In transient transfections, *IGFBP-3 *promoter activity increased in cells overexpressing HMGA1 and decreased in cells pretreated with siRNA detected against HMGA1. IGFBP-3 mRNA expression was higher in cartilage from patients with OA, where the increased expression of IGFBP-3 closely paralleled the increased expression of HMGA1 mRNA.

**Conclusions:**

Our observations indicate that increased HMGA1 expression in human chondrocytes is associated with increased expression of IGFBP-3. It is tempting to speculate that, through the regulation of IGFBP3 expression, HMGA1 may act as a pathogenetic factor for OA.

## Introduction

The high-mobility group A1 (HMGA1) protein is a small nuclear binding protein that participates in various fundamental cellular processes, including transcriptional regulation, embryogenesis, cell cycle regulation, transformation and differentiation, senescence, and DNA repair [1]. By interacting with AT-rich DNA binding sequences, HMGA1 is able to modify the structure of its target site and induce structural changes in the DNA to facilitate the progression of a wide range of DNA-dependent activities [2]. This interaction enables HMGA1 to recruit additional transcription factors and alter chromatin structure, leading to the formation of higher-order transcriptional complexes called enhanceosomes [3-5]. In concert with other factors, HMGA1 modulates gene expression and determines whether the associated genes are switched on or off [6]. Through such a mechanism, the transcription of cyclo-oxygenase-2, insulin-like growth factor (IGF)-binding protein-1 (IGFBP-1), and the insulin receptor is regulated by HMGA1 [4,5,7,8].

IGFBP-3 is a 38- to 43-kDa protein that regulates the bioavailability of IGF-I [9]. Detection of IGFBP-3 has been reported in human chondrocytes in articular cartilage [10,11]. Several cytokines, matrix metalloproteinases, and growth factors [10,12-15] take part in the regulation of articular cartilage homeostasis, and in developing articular cartilage, IGF-I is an inducer of collagen and aggrecan synthesis, representing an important factor in the maintenance of the chondrocyte phenotype as well as cartilage integrity and metabolism. During osteoarthritis (OA), normal or increased amounts of IGF-I are produced, but osteoarthritic chondrocytes are hyporesponsive to IGF-I; this property has been attributed to increased IGFBP levels that may interfere with IGF-I functionality [16-18].

The role of HMGA1 in OA has not been previously investigated. For the first time, we provide a comprehensive analysis of the molecular mechanisms underlying the activation of *IGFBP-3 *gene transcription in human chondrocytes. Using chromatin immunoprecipitation (ChIP) combined with studies of DNA-protein interaction and transient transfection assays in living cells, we demonstrate that HMGA1 modulates the transcription of the *IGFBP-3 *gene. Taken together, our findings provide a mechanistic insight into the molecular processes that influence *IGFBP-3 *gene expression in OA.

## Materials and methods

### Cells and protein extracts

HepG2 human hepatoma cells were cultured in RPMI 1640 medium supplemented with 10% fetal bovine serum. Because they naturally express both IGFBP-3 and HMGA1 protein species, these cells were used as a positive control model. T/C-28a4 and C-28/I2 cells (a kind gift from Mary B. Goldring, The Hospital for Special Surgery, New York, NY, USA, and Elizaveta Kon, Rizzoli Orthopedic Institute, Bologna, Italy) were cultured in high-glucose (4.5 g/L) Dulbecco's modified Eagle's medium supplemented with 10% fetal calf serum. Hepatoma, T/C-28a4, and C-28/I2 cell systems were supplemented with glutamine (2 mM), penicillin (100 IU/mL), and streptomycin (100 mg/mL) in a humidified atmosphere containing 5% CO_2 _at 37°C. Cytoplasmic and nuclear extracts were prepared as previously described [19], and the final protein concentration in the extract was determined according to the Bradford method (Bio-Rad Laboratories, Inc., Hercules, CA, USA).

### Oligonucleotides and electrophoretic mobility shift assay

Human *IGFBP-3 *gene promoter sequences were amplified from genomic DNA by polymerase chain reaction (PCR) by using the following specific primers: h1 forward, 5'-ATACAGTAATACGAAGTCGCC-3'; h1 reverse, 5'-ACCGGCAAGCGAATGCTCCTT-3'; h2 forward, 5'-TGGGGATATAAACAGCCCAGC-3'; and h2 reverse, 5'-ATACAGCGCTCCGCATTCGTG-3'. Fragments were end-labeled with ^32^P-dATP and used in an electrophoretic mobility shift assay (EMSA) as previously described [19].

### Chromatin immunoprecipitation

ChIP was performed as previously described [20] by using HepG2 cells. Formaldehyde-fixed DNA-protein complexes were immunoprecipitated with anti-HMGA1 antibody [19]. The following primers were used to amplify the region between -295 and -571 base pairs in the *IGFBP-3 *gene promoter: forward, 5'-TGGGGATATAAACAGCCCAGC-3' and reverse, 5'-GGTCACCCCAGTCACTCCT-3'. The PCR products were electrophoretically resolved on a 1.5% agarose gel and visualized by ethidium bromide staining.

### Plasmids and transfections

A human *IGFBP-3 *reporter plasmid (pGL3-*IGFBP-3 *Luc) was constructed by cloning the 1,876-base pair sequence of the human *IGFBP-3 *promoter (-1804/+72) into the pGL3 luciferase reporter vector (Promega Corporation, Madison, WI, USA) by using the Nhe I and Bgl II sites. The integrity of this construct was determined by DNA sequencing. pGL3-*IGFBP-3 *Luc was transiently transfected into cultured cells together with pcDNA3/HA-HMGA1 (a generous gift from Guidalbeto Manfioletti, University of Trieste, Trieste, Italy), pEVR2/Sp1 [4], and pSG5-C/EBPβ [4] vectors in the absence or presence of HMGA1 small interfering RNA (siRNA) (Santa Cruz Biotechnology, Inc., Santa Cruz, CA, USA) by using the Lipofectamine 2000 transfection reagent protocol (Invitrogen Life Technologies Corporation, Carlsbad, CA, USA). Luc activity was assayed between 48 and 96 hours later as previously described [21].

### Tissue preparation

The study protocol was approved by the local ethics committee, and the research was carried out in compliance with the Declaration of Helsinki. Informed written consent was obtained from all individuals. In total, 28 donors recruited at our outpatient clinic between January 2009 and January 2011 were grouped in two clusters. The first cluster consisted of 21 patients (10 women and 11 men with a mean age of 67.7 ± 7.7 years) who had OA (11 hip OA and 10 knee OA) and whose diagnosis was based on established clinical, biochemical, and radiological criteria [22,23]; all patients were subjected to total joint replacement. A radiological classification of each osteoarthritic joint was carried out according to the Kellgren-Lawrence [24] or Ahlbäck [25] scales. X-rays were independently evaluated by two musculoskeletal radiologists, who were unaware of the clinical characteristics of the patients, and the same measurements were repeated twice on two separate days. Cohen's kappa coefficients for interobserver and intraobserver reliability of scoring were 0.80 and 0.84, respectively. A consensus decision on the scores was reached in a final common readout, and a severe OA grade was noted in all patients (that is, five cases of grade III/IV hip OA, six cases of grade IV/IV hip OA, six cases of grade IV/V knee OA, and four cases of grade V/V knee OA). Patients with secondary OA or those who were previously subjected to intra-articular injections of corticosteroids, hyaluronic acid, or non-steroidal anti-inflammatory drugs were excluded from the study. The second cluster consisted of seven healthy donors (three women and four men with a mean age of 41.6 ± 10.7 years) with no OA or other diseases that may potentially alter the articular cartilage. Samples of articular cartilage were obtained as discarded surgical material in both clusters of patients. Specifically, cartilage was taken from the femoral head in patients with hip OA and from both femoral condyles and the tibial plateau in patients with knee OA. In the control group, the donor sites were the knee (three), hip (two), shoulder (one), and the proximal interphalangeal joint of the foot (one). All patients observed a pain medication-free interval 30 days prior to surgery. After the cartilage was removed from the joint, it was extensively washed with an isotonic saline solution to remove residual blood or synovial fluid. Specimens were excised from the superficial and deep layers of the articular cartilage by using disposable scalpel blades, avoiding the calcified layer and the subchondral bone. Approximately 0.5 cm^3 ^of cartilage was dissected from the rim of the ulcer present in all specimens from patients with OA, and an equal amount of tissue was taken far from the ulcer. This procedure of pooling together different samples of cartilage from the same joint overcomes, at least in part, the possible bias originating from the presence of non-homogenous osteoarthritic damage throughout the joint or the effect of a random choice of the tissue to be harvested. Samples were pooled, immediately immersed in liquid nitrogen, and stored at -80°C until analysis. Whole cartilage from healthy donors was similarly preserved.

### RNA preparation and qRT-PCR

Total cellular RNA was extracted from T/C-28a4 and C-28/I2 cultured cells by using the RNAqueous-4PCR Kit (Ambion, Austin, TX, USA). RNA isolation from cartilage samples was performed by using the TRI Reagent solution protocol (Ambion). The isolated RNA was subjected to DnaseI treatment, and cDNA was synthesized from 1 μg of total RNA. Both the yield of RNA and the 260/280 absorbance ratio were similar between samples obtained from osteoarthritic and healthy cartilage (data not shown). cDNA (10 ng) was used as a template in the quantitative real-time reverse transcription-PCR (qRT-PCR) reaction by using SYBR Green dye. Product accumulation was monitored with a Mastercycler ep Realplex (Eppendorf, Wesseling-Berzdorf, Germany) detection system. The mean cycle threshold (Ct) value was calculated from triplicate samples and used to determine the gene expression level. The sample ΔCt (SΔCt) value was calculated as the difference between the ribosomal protein S9 (RPS9) Ct and sample Ct values. The expression levels of type X collagen (ColX), HMGA1, and IGFBP-3 were expressed versus the control sample ΔCt (CΔCt) values. It has been previously described that ColX, the standard marker for chondrocyte hyperthophy [26], overexpresses in human osteoarthritic cartilage with respect to healthy adult articular cartilage [13,27-29]. Fold differences with respect to the control were determined by using the formula R = 2 - (SΔCt - CΔCt), where R represents a relative quantification. Target and housekeeping genes were amplified by using the following primers: COLXA1 forward, 5'-ACAGGAATGCCTGTGTCTGCTTTACT-3', COLXA1 reverse, 5'-CATTGGGAAGCTGGAGCCACACCTGGTC-3', HMGA1 forward, 5'-AGGAGCAGTGACCCATGCGT-3'; HMGA1 reverse, 5'-TGATGGTGGGCCTGGGGAAG-3'; IGFBP-3 forward, 5'-TTGCACAAAAGACTGCCAAG-3'; IGFBP-3 reverse, 5'-CAACATGTGGTGAGCATTCC-3'; and RPS9 forward, 5'-CAGCTTCATCTTGCCCTCAT-3' and RPS9 reverse, 5'-CTGCTGACGCTTGATGAGAA-3'.

### Statistical analysis

Student *t *test was used to assess the significance of the differences between values. The analysis of variance was used to evaluate the comparisons within the transient transfection assays. To calculate the power (1-β error probability; two-tailed) achieved by our statistical tests, we considered the actual sample size, the size of the observed effect, and the α value of 0.05. SPSS (version 17.0; SPSS Inc., Chicago, IL, USA) and G*Power (version 3.1; Institut für Experimentelle Psychologie, Heinrich Heine Universität, Dusseldorf, Germany) software were used for the database construction and the statistical analysis. A *P *value of less than 0.05 was considered significant.

## Results

### HMGA1 and IGFBP-3 expression in T/C-28a4 and C-28/I2 cells

**Figure 1:**
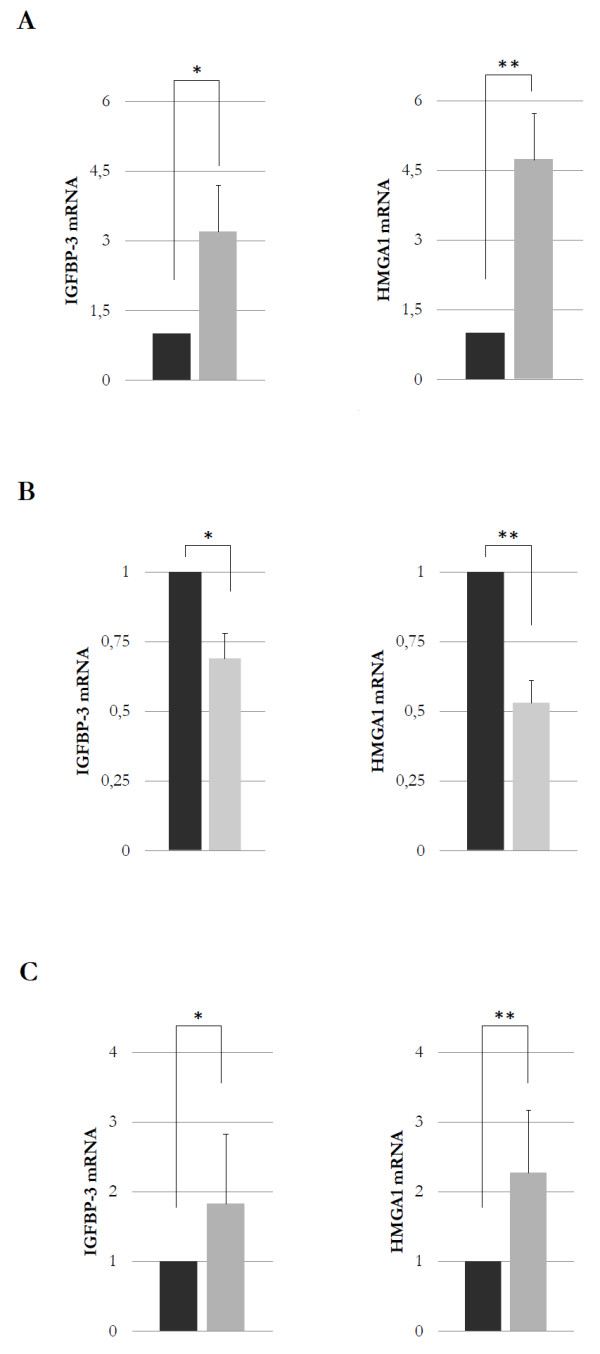
**IGFBP-3 and HMGA1 mRNA expression in chondrocytic cell lines**. **(a) **A comparison of IGFBP-3 and HMGA1 mRNA abundance is shown in T/C-28a4 (black columns) and C-28/I2 (gray columns) cells following qRT-PCR of total RNA from three separate experiments. *P = 0.0016; **P = 0.0005. **(b) **IGFBP-3 and HMGA1 mRNA as measured by qRT-PCR from cultured C-28/I2 cells pretreated with small interfering RNA directed against HMGA1 (gray columns) or untreated (black columns). *P = 0.0046; **P = 0.0004. **(c) **qRT-PCR of IGFBP-3 and HMGA1 mRNA in T/C-28a4 cells transfected with a pcDNA3/HA-HMGA1 expression vector (gray columns) or untransfected (black columns). *P = 0.0039; **P = 0.0031. Each column indicates the mean value; the error bars indicate standard deviation. HMGA1, high-mobility group A1; IGFBP-3, insulin-like growth factor binding protein 3; qRT-PCR, quantitative real-time reverse transcription-polymerase chain reaction.

As measured by qRT-PCR, IGFBP-3 and HMGA1 mRNA expression was observed in both T/C-28a4 and C-28/I2 cultured cell lines (Figure 1a). However, this expression was higher in C-28/I2 cells, and this may be consistent with the fact that these cells resemble primary chondrocytes more closely than T/C-28a4 cells do [30]. IGFBP-3 mRNA abundance in both cell lines closely paralleled HMGA1 mRNA production, suggesting that HMGA1 may play a role in *IGFBP-3 *gene activation. To examine further the role of HMGA1 in this event, we carried out experiments in C-28/I2 cells treated with siRNA directed against HMGA1. As shown in Figure 1b, a contextual significant decrease in both IGFBP-3 and HMGA1 mRNA levels was observed in C-28/I2 cells following transfection with HMGA1 siRNA. Conversely, an increase in IGFBP-3 expression was observed in T/C-28a4 cells following transfection with a pcDNA3/HA-HMGA1 expression plasmid that induced HMGA1 overexpression (Figure 1c). Taken together, these results indicate that a parallelism exists between IGFBP-3 and HMGA1 mRNA expression in chondrocytic cell lines.

### Physical interaction of HMGA1 with the *IGFBP-3 *promoter

**Figure 2:**
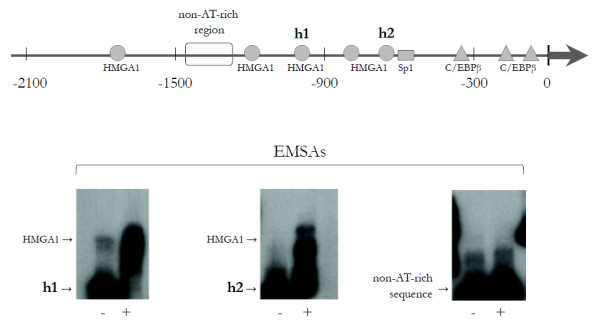
**Physical interaction of HMGA1 with the *IGFBP-3 *promoter**. In the upper panel, a schematic representation of the *IGFBP-3 *gene is shown with HMGA1, Sp1, and C/EBPβ DNA binding sites. h1 and h2 are *IGFBP-3 *DNA sequences that were used as probes. In the lower panel, EMSAs were performed by using the h1 and h2 DNA probes in the absence (-) and presence (+) of pure HMGA1 (5 ng). The non-AT-rich sequence was used as a negative control probe. C/EBPβ, CAAT-enhancer binding protein β; EMSA, electrophoretic mobility shift assay; HMGA1, high-mobility group A1; IGFBP-3, insulin-like growth factor binding protein 3.

**Figure 3:**
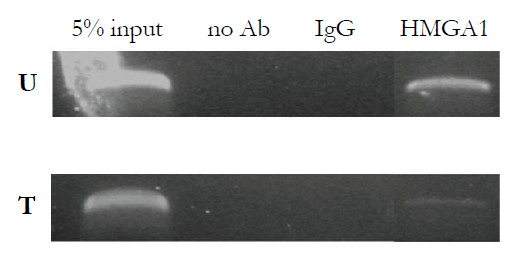
**ChIP of the *IGFBP-3 *gene promoter**. HepG2 cells, either untreated (U) or treated (T) with small interfering RNA directed against HMGA1, were used for ChIP of the *IGFBP-3 *gene by using a polyclonal antibody against HMGA1. There were three separate experiments. ChIP, chromatin immunoprecipitation; HMGA1, high-mobility group A1; IGFBP-3, insulin-like growth factor binding protein 3.

A schematic representation of the promoter region of the human *IGFBP-3 *gene is shown in Figure 2. Consensus transcription factor-binding sites within this region were explored by searching the TRANSFAC database by using MatInspector [31]. Putative binding sites for HMGA1 were identified together with consensus sites for the CAAT-enhancer binding protein β (C/EBPβ) and the ubiquitously expressed transcription factor Sp1. We focused on HMGA1 cis-sites surrounding the first 900 base pairs upstream of the transcriptional start site, which are usually associated with a more intense promoter activity [4]. DNA-HMGA1 protein interaction was characterized *in vitro *by EMSA by using the sequence-specific radiolabeled fragments h1 and h2, which include HMGA1 cis-sites flanking the Sp1 and C/EBPβ DNA binding motifs. As shown in Figure 2, binding of a pure recombinant HMGA1 isoform to these probes produced a protein-DNA complex that was recognized by the anti-HMGA1-specific antibody and was supershifted to a slower-migrating form. No DNA-HMGA1 protein interaction was observed when a non-AT-rich sequence of the *IGFBP-3 *gene promoter was used as a probe. The interaction of HMGA1 with the *IGFBP-3 *gene promoter was further explored in this study by ChIP analysis by using HepG2 cells, which naturally express HMGA1. As shown in Figure 3, binding of endogenous HMGA1 occurred at the *IGFBP-3 *gene locus in intact HepG2 cells. However, when HMGA1 expression was perturbed in HepG2 cells treated with siRNA directed against HMGA1, the DNA binding ability of HMGA1 was significantly reduced. This finding indicates that HMGA1 binds directly to the *IGFBP-3 *gene promoter and suggests a molecular mechanism for HMGA1-based stimulation of *IGFBP-3 *gene expression.

### HMGA1 is required for full activation of *IGFBP-3 *gene transcription

**Figure 4:**
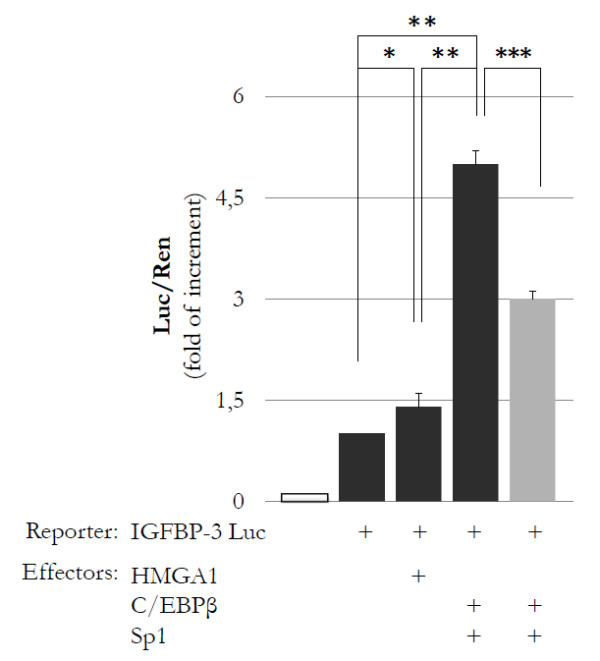
**Functional significance of HMGA1 for *IGFBP-3 *gene transcription**. HepG2 cells were transfected with the pGL3-*IGFBP-3 *Luc reporter plasmid (1 μg) in the presence of the indicated effector vectors (0.5 μg each, black columns). Empty column indicates mock (no DNA). Functional analysis with small interfering RNA directed against HMGA1 (100 pmol) is shown in the gray column. Transcriptional activity of the *IGFBP-3 *promoter is shown as the ratio of Luc activity to Renilla activity (Luc/Ren). Columns indicate mean values; error bars denote standard deviation. There were three separate experiments. *P = 0.03; **P < 0.0001; ***P < 0.0001. C/EBPβ, CAAT-enhancer binding protein β; HMGA1, high-mobility group A1; IGFBP-3, insulin-like growth factor binding protein 3.

Considering the observation that a physical interaction occurs between HMGA1 and the human *IGFBP-3 *promoter, we next investigated the functional significance of this DNA-protein interaction. To this end, HepG2 cells were transiently transfected with a human IGFBP-3-Luc reporter plasmid in the presence or absence of expression vectors containing HMGA1, Sp1, or C/EBPβ. As shown in Figure 4, IGFBP-3-Luc activity was enhanced in HepG2 cells overexpressing HMGA1. Induction of IGFBP-3-Luc activity markedly increased when both Sp1 and C/EBPβ were transfected simultaneously, implying that these nuclear factors function together to induce full *IGFBP-3 *gene transcription. Interestingly, the effect of Sp1 and C/EBPβ was blocked by treating cells with siRNA directed against HMGA1, confirming that HMGA1 plays a role in this process.

### HMGA1 and IGFBP-3 mRNA expression in patients with osteoarthritis

**Figure 5:**
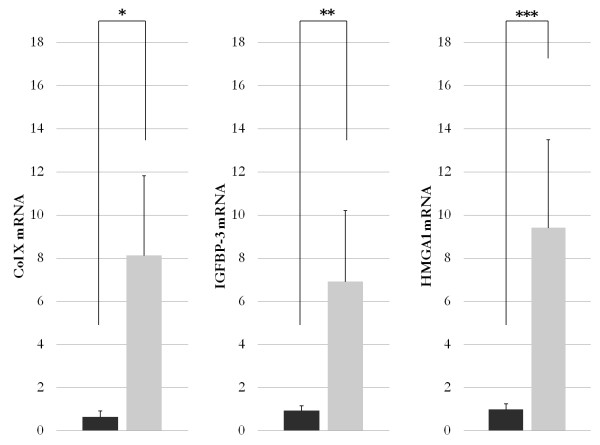
**ColX, IGFBP-3, and HMGA1 mRNA levels in osteoarthritis**. ColX (left), IGFBP-3 (center), and HMGA1 (right) mRNA expression in human chondrocytes from healthy donors (black columns) and from patients with osteoarthritis (gray columns), as measured by quantitative real-time reverse transcription-polymerase chain reaction of total RNA. Columns indicate mean values; error bars denote standard deviation. *P < 0.0001; **P = 0.0045; ***P = 0.0022. ColX, type X collagen; HMGA1, high-mobility group A1; IGFBP-3, insulin-like growth factor binding protein 3.

Increased IGFBP-3 protein expression has been previously reported in the cartilage of patients with OA [32,33]. Considering the above findings, which indicate that a functional relationship between HMGA1 and IGFBP-3 does indeed exist, we carried out studies in osteoarthritic human cartilage. As shown in Figure 5, IGFBP-3 mRNA expression, as measured by qRT-PCR, was significantly higher in cartilage from patients with OA compared with cartilage from healthy donors. Interestingly, the increased expression of IGFBP-3 in these patients closely paralleled the increased levels of HMGA1 mRNA, and this supports the hypothesis that HMGA1 may be responsible for IGFBP-3 overexpression in chondrocytes from patients with OA. Power analyses showed that the statistical tests used to compare ColX, IGFBP-3, and HMGA1 mRNA expression between cartilage from patients with OA and healthy donors had 99% power to detect the observed differences.

## Discussion

A key role for IGFBP-3 in modulating IGF-I bioavailability in human chondrocytes has been suggested previously [11,16-18,34]. However, the molecular mechanisms regulating expression in chondrocytes are still unknown. The possibility that HMGA1 plays a role in *IGFBP-3 *gene regulation is supported by the data in our present study, indicating that transactivation of the *IGFBP-3 *gene by the nuclear transcription factors Sp1 and C/EBPβ is dependent on HMGA1.

In our study, the functional link between HMGA1 and IGFBP-3 was initially evaluated by molecular biological techniques and gene expression analyses in both T/C-28a4 and C-28/I2 chondrocytic cultured cells, which are two *in vitro *cell models that are commonly used for studies on articular cartilage pathophysiology [30]. Our results clearly indicate that HMGA1 positively modulates *IGFBP-3 *gene expression in both chondrocyte cell lines. This finding is in apparent contrast to previous observations indicating that downregulation of the *IGFBP-3 *gene by HMGA1 occurs in mouse C2C12 cells that stably overexpress HMGA1 [35]. An explanation for this apparent discrepancy could be that the sustained induced overexpression of HMGA1 in C2C12 cells may severely preclude IGFBP-3 expression by preventing myoblast differentiation. This possibility is supported by the fact that the expression of both myogenin and MyoD, two key players in muscle cell differentiation [36,37], is abolished in C2C12 cells constitutively expressing HMGA1. Furthermore, the cell specificity of HMGA1 action should be considered. In this respect, a differential role of HMGA1 as either an activator or an inhibitor of cellular biological processes has been documented [1]. The *in vitro *data in cultured chondrocytes were confirmed in articular cartilage samples from patients with OA. In agreement with previous studies [32,33], IGFBP-3 levels were increased in the cartilage of patients with OA, and a parallel increase in HMGA1 expression was detected simultaneously. To the best of our knowledge, this latter observation has not been reported before, and the increase in HMGA1 expression in these patients constitutes a novel finding in this study. We next observed an overexpression of type X collagen in osteoarthritic cartilage. However, caution should be exerted in interpreting this overexpression because conflicting results have been published for the expression of this collagen type in OA [26].

Autocrine IGFBP-3 production in intact cartilage has been reported [32]. Notably, the increase of IGFBP-3 during OA may affect the integrity of articular cartilage, diminishing the synthesis of matrix collagen and aggrecan [9,13].

IGF-I functions in the stimulation and maintenance of the chondrocyte phenotype principally in immature cartilage [38]. Therefore, the reduced IGF-I bioavailability in OA might influence chondroptosis, thus creating cell-depleted cartilage [39-41]. An increase in IGFBP protein production has been widely demonstrated in both the articular cartilage and synovial fluid from patients with OA [16,34,42,43], and the increased amount of IGFBP-3 has been directly associated with the severity of the disease [32]. IGFBP-3 overexpression has been reported in the extracellular cartilage matrix and within both the cytoplasm and nuclear environment of osteoarthritic chondrocytes [11]. Cellular segregation of IGFBP-3 might also deliver IGF-independent signals [34]. Notably, the nuclear presence of IGFBP-3 in chondrocytes has been associated with the regulation of cell division and apoptosis [11]. However, the possibility that HMGA1 plays a direct role in chondroptosis cannot be excluded, and this possibility is consistent with data in the literature, indicating that apoptosis in non-neoplastic cells can be induced by HMGA1 [44]. Finally, a possible role for HMGA1 and IGFBP-3 in response to OA-induced hypoxia can be hypothesized. Indeed, hypoxia is involved in the pathogenesis of several musculoskeletal diseases [45,46] and is able to induce IGFBP-3 via HIF-1α in a wide range of cell cultures [47,48]. It has also been demonstrated that hypoxia increases the expression of HMGA proteins [7].

## Conclusions

Collectively, our data indicate that IGFBP-3 expression is increased both in the C-28/I2 chondrocytic cell line and in the chondrocytes of osteoarthritic patients in comparison with T/C-28a4 cell line and healthy human cartilage controls, respectively. Given our observations that HMGA1 is implicated in the upregulation of IGFBP-3 protein expression, it is tempting to hypothesize that HMGA1 acts as a regulator of articular cartilage homeostasis. Future studies will further clarify the role of HMGA1 in the pathomechanisms of OA.

## Abbreviations

C/EBPβ: CAAT-enhancer binding protein β; ChIP: chromatin immunoprecipitation; ColX: type X collagen; Ct: cycle threshold; EMSA: electrophoretic mobility shift assay; HMGA1: high-mobility group A1; IGF-I: insulin-like growth factor I; IGFBP-3: insulin-like growth factor binding protein 3; OA: osteoarthritis; PCR: polymerase chain reaction; pGL3-*IGFBP-3 *Luc: human *IGFBP-3 *reporter plasmid; qRT-PCR: quantitative real-time reverse transcription-polymerase chain reaction; RPS9: ribosomal protein S9; siRNA: small interfering RNA.

## Competing interests

The authors declare that they have no competing interests.

## Authors' contributions

GG, AB, and OG conceived the study, had full access to all of the data, and wrote the manuscript. MDG, FP, and EC performed most of the experiments and acquired the data. OG made a critical revision of the manuscript for important intellectual content. All authors have read and approved the final manuscript.

## Authors' information

GG has an MD and is a full professor of orthopedics and head of the Orthopedics and Traumatology Department, Magna Græcia University and Mater Domini University Hospital (Catanzaro, Italy). MDG has an MD and is a resident in orthopedics and traumatology, Magna Græcia University and Mater Domini University Hospital. FP has a BS and is a researcher at the Department of Neuroscience and Brain Technologies, Istituto Italiano di Tecnologia (Genoa, Italy). EC has an MD and is a researcher at the Department of Health Sciences, Magna Græcia University. AB has an MD and a PhD and is an associate professor of endocrinology, Department of Health Sciences, Magna Græcia University and Mater Domini University Hospital. OG has an MD and is an assistant professor of orthopedics, Department of Orthopedics and Traumatology, Magna Græcia University and Mater Domini University Hospital.
